# Effect of lifestyle modification for two years on obesity and metabolic syndrome components in elementary students: A community- based trial

**DOI:** 10.22088/cjim.13.3.555

**Published:** 2022

**Authors:** Bita Rabbani, Hossein Chiti, Faranak Sharifi, Saeedeh Mazloomzadeh

**Affiliations:** 1 Zanjan Metabolic Diseases Research Center, Zanjan University of Medical Sciences, Zanjan, Iran; 2 Zanjan Social Determinants of Health Research Center, Zanjan University of Medical Sciences, Zanjan, Iran

**Keywords:** Metabolic syndrome, Obesity, Life style, Nutrition, Hypertension

## Abstract

**Background:**

Lifestyle modifications, especially improving nutritional patterns and increasing physical activity are the most important factors in preventing obesity and metabolic syndrome in children and adolescents. For this purpose, the following interventional study was designed to investigate the effects of educational programs for students, as well as the changes in diet and physical activity on obesity and components of the metabolic syndrome.

**Methods:**

This study is part of an interventional research project (elementary school) conducted on all students of Sama schools in Zanjan and Abhar in three levels;elementary, middle and high school, including 1000 individuals in Zanjan (intervention group) and 1000 individuals (control group) in Abhar in 2011. Interventions were based on educating students, teachers and parents, changes in food services and physical activity. We primarily measured anthropometric indices, fasting blood sugar, lipid profiles and blood pressure and completed standard nutrition and physical activity questionnaires. Also, blood insulin levels were randomly measured in a number of students. Data analysis was done by SPSS software Version 16.0.

**Results:**

Overall, 589 individuals (252 males, 337 females) entered the case group and 803 individuals (344 males, 459 females) entered the control group. After two years of intervention, the mean waist circumference (63.8±10.9) and diastolic BP (63.8±10.4) were significantly lower, however, the mean systolic BP (10.1.0±12.5), food score (25.0±5.0) and drinking score (12.1±2.3) were higher in the intervention group (p<0.001). Comparing the components of metabolic syndrome between the second year and at the time of recruitment within the intervention group, showed that although the number of overweight/obese individuals, individuals with hypertriglyceridemia and high LDL increased, while those with abdominal obesity, high BP, hyperglycemia, and insulin resistance decreased (p<0.001). On the other hand, in the control group, the number of individuals with high BP increased significantly.

**Conclusion:**

The prevalence of abdominal obesity and hypertension, which are the two major components of metabolic syndrome, are much higher in our study than the other regions of the country. However, interventions for modification of diet and increasing physical activity, are effective in lowering of their prevalence.

Childhood obesity as one of the criteria for diagnosing metabolic syndrome has become a global health problem in recent decades. The prevalence of metabolic syndrome (MeS) in children has increased in recent decades (1).

The NCEP Adult Treatment Panel III (ATP III) as one of the most important sources of MeS definitions, have been mentioned to high blood pressure, dyslipidemia, high blood sugar, and insulin resistance, in addition to abdominal obesity as criteria for diagnosing this syndrome, all of which increase the morbidity and mortality of cardiovascular disease (2). ATPIII criteria modified for age describes the MetS presence when three of the following criteria are met: systolic blood pressure (BP) or diastolic BP ≥90th percentile for age and gender, triglyceride (TG) ≥110 mg/dl, HDL-C ≤40 mg/dl, waist circumference (WC) ≥90th percentile for age and gender and fasting glucose (FG ) ≥110 mg/dl (3).

Based on a meta-analysis, school-based programs were able to reduce the prevalence of overweight and obesity (4). Some studies showed no reduction in body mass index (BMI) compared to a control group, while others have shown that nutritional interventions and physical activity have a significant effect on BMI (4, 5). Based on a meta-analysis, school-based programs were able to reduce the prevalence of overweight and obesity (5). Some studies have shown no reduction in body mass index (BMI) compared to a control group, while others have shown that nutritional interventions and physical activity have a significant effect on BMI (5, 6). Many disease processes start at a young age including atherosclerosis, which then lead to cardiovascular diseases (6, 7). Primary school children seem to be particularly sensitive to the behaviors which are taught to them. For example, Magriplis et al. demonstrated that sleep, family meals, and study hours were all positively correlated with dietary patterns, while total screen time, frequency of dining out, and eating while watching television were all negatively linked. Interventions to decrease and prevent childhood obesity will almost certainly require to address particular behavioral and nutritional patterns in combination (8). Vogeltanz-Holm et al. showed that an effective intervention for lowering or maintaining BMI in rural White and American Indian kids is a primary school health care program that targets a variety of children' obesity-related health behaviors, the school health milieu, and engages parents and educators (9).

One of the most commonly established metabolic syndrome criteria is the NCEP ATP III definition. It includes visceral obesity, hyperglycemia/insulin resistance, hypertension and atherogenic dyslipidemia as main characteristics. Based on a meta-analysis, school-based programs have been able to reduce the prevalence of overweight and obesity. Some studies showed no reduction in body mass index (BMI) compared to a control group (5), while others showed that nutritional interventions and physical activity had a significant effect on BMI (4).

The efficacy of school-based interventions can be impacted by a variety of factors beyond the researchers' control. While the period of the program and the intervention utilized should be clearly mentioned in the research design, variables such as participant age, school environment, parental engagement, and collaboration with the researchers are not completely controlled (10). An analysis on intervention period, showed that programs that last for more than a year, have a significantly stronger impact on changes in BMI than those with a duration of less than 6 month. In other words, the longer the intervention, the higher number of participants will experience weight loss. In addition, interventions that can be used to prevent weight gain aside to an increase in height and fat mass can lead to a decrease in BMI (5). However, evidence on impact of school-based programs on reducing overweight and obesity in the short or long term is not completely understood. In this study, for the first time, we aimed to evaluate the effect of lifestyle modification interventions in a community-based trial on components of MeS among elementary school children. In here we report the two-year findings in our community trial. 

## Methods

**Study settings:** In this multi-centered cohort field study, include 1000 individuals in Zanjan (intervention group) and 1000 individuals in Abhar (control group), all the students from Sama elementary schools (589 and 803 individuals as case and control groups, respectively) during 2011 participated in the study (Supp 1). The reason for choosing Sama schools, is their single coordinated management which allowed the implementation of study protocol in all schools. The students of these schools have a medium to high socioeconomic class. The study protocol and interventional programs were approved by the Institutional Review Board of Zanjan University of Medical Sciences, Zanjan, Iran (Ethics code no ZUMS.REC.1394.190).

**Research group:** The medical research team included three specialists in the field of endocrinology and metabolism, four pediatricians, three general practitioners and a team of nurses, nutritionists and experts in the field of research. General practitioners, and nurses underwent training regarding study protocol, measurement of height, weight, abdominal circumference, blood pressure, and body mass index (BMI) and the study questionnaires. All related training was under the supervision of a specialist in endocrinology, and errors in measurements were resolved. In this study, only children with a pre-existing underlying disease who were unable to cooperate with the researchers were excluded at the beginning of the study.

**Measurements: **At the beginning of the study, a preliminary study was conducted to determine the knowledge, attitude and nutritional status of students using two questionnaires from the pathway study (11) among elementary school students. Furthermore, physical activity of students was evaluated by a questionnaire from the pathway study. At the beginning of the study, all of the students in the intervention and control groups were evaluated regarding weight and height, BMI, blood pressure and abdominal circumference. After 12 hours of fasting, all participants had blood tests for fasting blood sugar (FBS), cholesterol, TG, HDL cholesterol and LDL cholesterol. Moreover, insulin levels were also evaluated and recorded. Blood samples were taken in a sitting position from the cubital vein. All tests were performed after centrifugation. Serum isolation of the samples was done in Valiasr Hospital in Zanjan, Iran. All measurements were repeated at the end of each school year for 3 years. MeS was defined according to the National Cholesterol Education Program’s Adult Treatment Panel (ATP) III criteria (12) and based on the presence of central obesity and BMI more than the 90^th^ percentile, with at least two of the following criteria: TG ≥ 150mg/dl, HDL-c < 40mg/dl, systolic blood pressure > 130mmHg or diastolic blood pressure > 85mmHg and FBS > 100mg/dl. The reason for selecting the mentioned school sections for laboratory testing was greater assurance in the presence of student during the three-year study period to evaluate intervention results. In the absence of a relevant student at the school, another classmate who had attended the school for at least three years was randomly invited to join the study.

**Attitude and nutritional practice:** A questionnaire adapted from the pathway study was used to assess students' attitudes and performance. Validity was based on Expert opinion and its reliability was measured during a pilot study in 2010 on 20 students. After obtaining an 80% reliability the questionnaires were used for the study. The questionnaires had 16 questions, 11 of which focusing on students’ attitude on important nutritional issues and 5 questions focusing on their daily performance with tangible examples of food choices in their everyday life. The total points of the questionnaires would vary from 0 to 43, and those with lower than 24 would be classified as weak and those with higher than 24 as good (supplement 1).

Students filled another questionnaire about their drinking performance status that had 8 questions and each question had 2 points. Scores less than 5 would be considered inappropriate, between 5 and 10 was considered medium and higher than 10 as appropriate (supplement 2).

Parents were educated on how to fill the questionnaires by the cohort team in 3 sessions and students would complete their questionnaires at home with the help of their parents. Students could use the colored charts that were designed at the end of the questionnaires to evaluate their points and to evaluate their status regarding their nutritional habits and drinks. 

The Food Habits Questionnaire (FHQ) is a 20- item self-report questionnaire that measures food intake habits. Questions are about typical eating patterns over the past month, and are rated on a 4-point Likert scale from 1 “Never or rarely” to 4 “Usually or Always”, or “Not applicable”. Fat intake is assessed by focusing on four food selection behaviors: excluding high-fat ingredients and high-fat cooking methods, choosing specially manufactured low-fat food products instead of high-fat ones, replacing high-fat foods with low-fat substitutes, and modifying high-fat foods.

**Physical activity status survey:** Physical activity status of students was measured using a standard questionnaire from the pathway study. Validity and reliability were measured and validated at the beginning of the study in the pilot study on 30 students (supplements 3 and 4). In this questionnaire, activities were evaluated separately during school days and summer holidays, and based on the special scoring system that weighs the questions, 2 types of scores including Training Stress Score (TSS) and Tegner Activity Scale (TAS) were calculated and the total annual physical activity of each individual was estimated quantitatively. TSS is a composite number that is taken into account the duration and intensity of a workout to arrive at a single estimate of the overall training load and physiological stress created by that training session. Also, TAS aims to provide a standardized method in determining the level of activity prior to injury and level of activity post injury that can be documented on a numerical scale.

**Anthropometric measurements:** Anthropometric measurements were done by a team of five in Zanjan city and another team of five in Abhar. To measure height, a wall stadiometer (Seca,Germany), with a 0.5cm error, was used. For the measurement of weight scale (Seca,Germany), a precision of 0.1kg, was used. Measuring the weight of the students was done with minimal clothing. Abdominal circumference measurement was done without clothing from the umbilicus, using a standard tape measure with an accuracy of 0.1cm. Regarding BMI, those with a percentile above 95 were considered obese and those with a percentile of 95-85 were considered overweight (13).

**Blood pressure measurement:** Blood pressure was measured with a digital measuring barometer (ALPK2, Japan) with an accuracy of 0.1mmHg. All blood pressures were taken from the left arm in a sitting position using appropriate cuffs. If high blood pressure was detected, it was checked again at intervals of 10 minutes three times and the average was considered as that individual’s blood pressure. In this study, systolic and diastolic blood pressures higher than the 95^th^ percentile were considered hypertension and between the 90^th^ and 95^th^ percentiles were considered prehypertension (13).

**Laboratory measurements:** Students were randomly selected and invited to the Metabolic Disease Research Center affiliated to Zanjan University of Medical Sciences, Zanjan, Iran and the Abhar Health Center in Abhar, Iran for their blood tests. In Zanjan, all measurements were carried out on the same day in the laboratory of Valiasr Hospital by a trained expert. Blood tests in Abhar, were done in Abhar Central Laboratory with similar kits to that used in Zanjan. The remaining serum of individuals were immediately frozen in -20 centigrade and were sent weekly to Valiasr Hospital lab, Zanjan, Iran with cold chain observation for relevant measurements such as insulin levels. Insulin was measured using the ELISA method using "mono-bind" kits. Insulin resistance "HOMA-IR" was calculated using the following formula: 

Fasting insulin (μU/L)*fasting glucose (nmol/L)/22.5

Values above 2.1 were considered insulin resistance (14).

**Interventions:** The key elements of interventions were developed by a committee consisting of a researcher, the principals of Sama schools and representatives of the Roozbeh Institute who were responsible for catering the schools. The interventions in this study were based on five pillars: educating students in classrooms, changes in buffets and food services, student physical activity, teacher education, and parental education.

**Educating students in classrooms:** Students' formal education was carried out by pediatricians focusing on the importance of obesity and basic principles of proper nutrition during two, two-hour sessions per academic year and a total of 12 hours for each student. In addition, the researcher and his colleagues developed a booklet for the students and their parents and distributed it at the end of the first year of the interventions. To encourage students to read the book and understand the implications of those concepts, competitions were held and students were presented with questions that were answered in the booklet, and awards were given. Moreover, content of this booklet also included calculation of BMI, finding the status of each student on the growth curves and calculating calorie consumption, which was taught to students by the researcher during the two-hour sessions.

**Physical Activity:** The main goal was to perform moderate to severe physical activity for at least 30 minutes three times a week. For this purpose, interventions were designed on physical activity of students by convening meetings between the researcher and gym teachers of all schools involved in the intervention. Increasing students' awareness on the benefits of physical activity was part of the contents in theoretical classes. Another strategy was to change the time of morning exercises, so that the majority of students participate, as well as increasing its duration which was done by receiving approval from the school principals. A booklet containing various games was developed for gym teachers with the assistance of the Zanjan Physical Education Organization and was available for all teachers to increase physical activity of students during gym classes.

In addition, the educational CD provided teachers and students with knowledge about aerobic activities and asked teachers to participate in morning exercises alongside the students. Strengthening school gyms and organizing standard sport halls for the use of students during gym hours was another of the actions taken in co-ordination with the management of Sama schools. Encouraging students to increase their physical activity at home was another point that was considered. Encouraging the reduction of time spent watching TV and gaming to less than 4 hours a day, using stairs instead of elevators, doing chores, and promoting traditional (involving physical activity) games was another step that was emphasized during classes for students and their parents. 

**Teachers' participation in intervention:** Involving teachers in the Sama intervention project was one of the main strategies of the project, because most students followed their teachers as a role model. For the teachers, a two-hour session of education was held by the researcher, while emphasizing the importance of correct education, their participation in planning and promoting lifestyle improvement in students was sought. Teachers, alongside students, used the new nutritional models. This aided in better participation of students, better reporting, increased students' engagement in health issues, and aided in better participation in health promotion activities such as artistic assignments (painting, writing, and etc.). Developing a student newspaper focusing on healthy eating and holding a competition was one of the steps taken to encourage cooperation of students. Repeating teachers' recommendations regarding the disadvantages of consumption of salt during classrooms as well as promoting paintings or posters on this matter were some of the other actions taken. The health sponsor scheme was attended by student volunteers and a health assistant was chosen from the students. Health assistants trained other students to use proper nutrition.

**Parental education:** At the beginning of the project in 2011, two-hour sessions were held at the schools with the participation of students’ parents. During which, the researcher, using slides, tried to justify the importance of obesity and its role in the health of students. Throughout the school year, educational sessions were held for parents, especially mothers, who have a major role in providing food at home, and they were educated on proper nutrition. Parents were educated regarding ways to increase student physical activity, the use of bicycle for transportation, selection of foods, and reducing time spent on watching television and computer games to less than 4 hours a day. At the end of the first year of intervention, parents were informed about the results of students' tests and also the results of the statistical analysis of the first stage of study. Additionally, parents were made aware of their participation in changing the students’ lifestyle at home. Parents of elementary school students also played a major role in completing questionnaires and taking blood samples for testing.

**Nutrition services in schools:** Since 2011, starting with the Sama project, all school canteens were cleared of unhealthy food, and according to the protocol provided by the Sama policy council, only healthy products were delivered. Healthy foods, as meals, were delivered to students in all the schools that were part of the intervention group. Healthy snacks were prepared by the Roozbeh Institute in Zanjan, Iran and under the supervision of a nutrition expert, by calculating the amount of calorie intake and by increasing the intake of legumes and fiber and reducing fat and salt content in children's food. The nutritionists' further made changes in the nutritional content of the foods, while preserving color and taste of foods. Each day, these snacks were distributed among the students at certain times, and students were only allowed to have fruits in between these snacks. Promoting the use of healthy fluids was done by placing mineral water in buffets and removing drinks containing high carbohydrates and carbonated beverages from the school canteens. Encouraging students to use fruits instead of juice was another step taken. Buffets served the role of preparing warm snacks using beans and vegetables under the supervision of a nutritional expert. Students were given a choice of having warm food or other healthy foods in the school canteen.

**Protocol evaluation:** School principals were responsible for monitoring the implementation of interventions at the schools. When facing problems with the implementation of interventions, the researcher or the project manager would be contacted and problems would be acknowledged in meetings and solutions would be discussed. The nutrition expert was responsible for checking the food catering, calculating calories and content of the snacks in accordance to research objectives, by randomly checking different schools during the week and giving out reports on the project. A physical education specialist was responsible for programs related to physical activity of students, who would monitor the implementation of programs during the study. During the interventions, once a year, interviews were conducted with parents and students by the project manager using a focus group method and all of their views were evaluated. At the end of each academic year, a report on the progress of the project was presented to the members of the policy council by the project manager, and the main policies were adopted in the new academic year.

**Data gathering:** Data entry and completing the questionnaires, were done by one person for each school, either the gym instructor or the health instructors. Training sessions for these coaches were done by the facilitator and they were all matched in the measurements.

**Data analysis:** Data were analyzed using the Statistical Package for Social Sciences (SPSS Inc., Chicago, IL, USA), SPSS for windows, Version 20. The independent sample t-test was used for comparing the mean of quantitative variables with normal distribution between two groups and for comparison of quantitative variables without normal distribution between two groups, the Mann-Whitney test was used. For comparison of pre- and post-intervention variables with normal distribution the paired t-test and for comparison of variables without a normal distribution, the Wilcoxon test was utilized. Qualitative data were compared between groups using the chi-square test. A p-value of less than 0.05 was considered statistically significant.

## Results

In 2011, a total of 1392 elementary students entered the study. Overall, 589 individuals were included in the case group [252 (42.7%) males and 337 (57.3%) females] from Zanjan city and 803 [459 (57.2%) females and 344(42.8%) males] individuals were included in the control group from Abhar city. Table 1 shows a comparison of baseline and anthropometric characteristics between the intervention and control groups at the beginning of the study and in two years of follow-up. Results showed that those in the case group had significantly higher BMI, systolic and diastolic BP, lower food score, higher appropriate drinking score, lower TSS and lower TAS at beginning of the study (p<0.05). Overall comparison of components of MeS between the intervention and control groups at the beginning of the study showed that number of overweight and obese individuals, individuals with high systolic blood pressure and those with high insulin resistance were significantly higher in the intervention group (table 2).

**Table 1:** Comparison of anthropometric indices, nutrition and physical activities of primary school students between intervention and control groups at the beginning of the study and in two years*

**Variables**		**Groups**		**P-value**
		**Intervention**	**Control**	
Height – cm	Beginning of study	126.0±12.6	134.6±10.9	<0.001
	End of second year	134.2±10.4	143.0±10.9	<0.001
Weight - kg	Beginning of study	29.9±9.2	31.5±9.5	0.002
	End of second year	32.3±10.3	36.2±11.2	<0.001
BMI - kg/m^2^	Beginning of study	18.6±3.5	17.1±3.2	<0.001
	End of second year	17.6±3.7	17.4±3.6	0.229
Abdominal circumference – cm	Beginning of study	61.2±9.5	61.6±9.5	0.32
	End of second year	63.8±10.9	66.8±10.4	<0.001
Systolic blood pressure - mmHg	Beginning of study	103.1±12.6	95.3±10.2	<0.001
	End of second year	101.0±12.5	98.6±6.5	<0.001
Diastolic blood pressure - mmHg	Beginning of study	65.6±12.1	62.4±4.4	<0.001
	End of second year	64.9±10.4	66.4±5.9	0.001
Food habit points	Beginning of study	23.2±4.9	24.2±4.9	0.002
	End of second year	25.0±5.0	23.9±5.4	0.001
Healthy drinking points	Beginning of study	12.02±2.5	11.4±2.3	<0.001
	End of second year	12.1±2.3	10.9±2.5	<0.001
Inactivity points (TSS)	Beginning of study	2.91±1.6	3.3±1.7	<0.001
	End of second year	3.2±1.7	3.5±2.0	0.155
Physical activity points (TAS)	Beginning of study	5.8±2.6	6.5±2.4	<0.001
	End of second year	6.7± 2.6	7.1±3.2	0.176

BMI: Body mass index; TSS: Training Stress Score; TAS: Tegner Activity Scale *All plus-minus values are means and standard deviations.

After two years of intervention, comparison of the two groups showed that mean WC and diastolic BP were significantly lower in the intervention group, however mean systolic BP, food score and drinking score were significantly higher in the intervention group (p<0.001). Moreover, comparison of components of MeS between the two groups in the second year showed that number of individuals with obesity and over-weight individuals, number of individuals with high BP, and total number of individuals with MeS was significantly lower in the intervention group compared to the control group (p<0.001) (table 3). 

**Table 2:** Comparison of metabolic syndrome components between intervention and control groups at the beginning of the study.*

**Variables**		**Groups**		**P-value**
		**Intervention**	**Control**	
Abdominal obesity – no. (%)	Males	11 (4.5)	12 (4.9)	0.870
	Girls	21 (6.1)	15 (4.4)	0.296
	Both	34 (5.8)	27 (4.6)	0.330
Overweight – no. (%)	Males	55 (22.8)	35 (14.1)	<0.001
	Females	60 (17.6)	46 (13.4)	<0.001
	Both	115 (19.7)	81 (13.7)	<0.001
Obesity – no. (%)	Males	101 (41.9)	39 (15.7)	<0.001
	Females	55 (16.1)	25 (7.3)	<0.001
	Both	158 (27.1)	64 (10.8)	<0.001
High blood pressure – no. (%)	Males	155 (64.3)	216 (87.4)	<0.001
	Females	286 (84.4)	224 (65.5)	<0.001
	Both	443 (76.4)	440 (74.7)	0.505
High systolic blood pressure – no. (%)	Males	101 (41.9)	105 (42.5)	0.893
	Females	238 (70.2)	77 (22.5)	<0.001
	Both	340 (58.5)	182 (30.9)	<0.001
High diastolic blood pressure – no. (%)	Males	137 (56.8)	211 (85.8)	<0.001
	Females	272 (80.2)	214 (62.6)	<0.001
	Both	411 (70.7)	425 (72.3)	0.592
Hypertriglyceridemia– no. (%)	Males	5 (5.5)	1 (3.3)	0.0636
	Females	8 (6.7)	1 (2.3)	0.269
	Both	13 (6.2)	2 (2.7)	0.249
LDL ≥ 110mg/dL – no. (%)	Males	15 (16.5)	0 (0)	0.018
	Females	19 (16.0)	12 (27.3)	0.102
	Both	34 (16.2)	12 (16.2)	0.996
Low HDL– no. (%)	Males	28 (30.8)	8 (26.7)	0.670
	Females	41 (34.5)	11 (25.0)	0.250
	Both	69 (32.9)	19 (25.7)	0.251
Hyperglycemia (>100mg/dL) – no. (%)	Males	2 (1.5)	0 (0)	0.502
	Females	6 (5.0)	0 (0)	0.129
	Both	8 (3.1)	0 (0)	0.122
Insulin resistance – no. (%)†	Males	16 (17)	2 (7.4)	0.216
	Females	42 (35.6)	3 (6.8)	<0.001
	Both	58 (27.4)	5 (7.0)	<0.001
Metabolic syndrome – no. (%)	Males	4 (4.1)	0 (0)	0.269
	Females	9 (7.7)	1 (2.3)	0.204
	Both	13 (6.5)	1 (1.4)	0.105

*Components of metabolic syndrome are defined according to the Program’s Adult Treatment Panel (ATP) III criteria as TG ≥ 150mg/dl, HDL-c < 40mg/dl, systolic blood pressure > 130mmHg or diastolic blood pressure > 85mmHg and FBS > 100mg/dl. †Insulin resistance was defined as insulin resistance index (HOMA-IR) greater than 1/2.

**Table 3:** Comparison of frequency distribution of metabolic syndrome components between intervention and control groups among elementary students at end of the second year.*

**Variables**		**Both groups**		**P-value**
		**Intervention**	**Control**	
Abdominal obesity – no. (%)	Males	5 (2.2)	10 (4.4)	0.189
	Females	8 (2.9)	9 (2.8)	0.907
	Both	13 (2.6)	19 (3.4)	0.400
Overweight – no. (%)	Males	13 (5.8)	35 (5.8)	0.029
	Females	20 (7.2)	15 (4.6)	0.194
	Both	39 (7.8)	52 (9.4)	0.400
Obesity – no. (%)	Males	12 (5.3)	28 (12.4)	
	Females	27 (9.8)	24 (7.4)	
	Both	33 (6.6)	28 (5.1)	
High blood pressure – no. (%)	Males	79 (35.0)	150 (66.4)	<0.001
	Females	133 (48.4)	231 (71.7)	<0.001
	Both	212 (43.3)	381 (69.5)	<0.001
High systolic blood pressure – no. (%)	Males	38 (16.8)	34 (15.0)	0.607
	Females	84 (30.5)	53 (16.5)	<0.001
	Both	122 (24.4)	87 (15.9)	0.001
High diastolic blood pressure – no. (%)	Males	76 (33.6)	150 (66.4)	<0.001
	Females	116 (42.2)	231 (71.7)	<0.001
	Both	192 (38.3)	381 (69.5)	<0.001
Hypertriglyceridemia – no. (%)	Males	14 (21.9)	2 (8.3)	0.142
	Females	29 (36.2)	1 (2.3)	0.269
	Both	43 (29.9)	6 (13)	0.020
LDL >=110 mg/dL – no. (%)	Males	17 (26.6)	4 (16.7)	0.332
	Females	34 (42.5)	8 (36.4)	0.605
	Both	51 (35.4)	12 (26.1)	0.240
HDL– no. (%)	Males	21 (32.8)	3 (12.5)	0.057
	Females	15 (18.8)	7 (31.8)	0.187
	Both	36 (25)	10 (21.7)	0.650
Hyperglycemia– no. (%)	Males	1 (1.6)	0 (0)	0.538
	Females	0 (0)	0 (0)	1.000
	Both	1 (7.0)	0 (0)	0.570
Insulin resistance – no. (%)	Males	6 (9.5)	5 (20.8)	0.156
	Females	35 (45.5)	3 (13.6)	0.007
	Both	41 (29.3)	8 (17.4)	0.112
Metabolic syndrome – no. (%)	Males	10 (23.3)	0 (0)	0.049
	Females	4 (7.3)	2 (15.4)	0.354
	Both	14 (143)	2 (6.9)	0.300

*Components of metabolic syndrome are defined according to the Program’s Adult Treatment Panel (ATP) III criteria as TG ≥ 150mg/dl, HDL-c < 40mg/dl, systolic blood pressure > 130mmHg or diastolic blood pressure > 85mmHg and FBS > 100mg/dl. †Insulin resistance was defined as insulin resistance index (HOMA-IR) greater than 1/2.

At the beginning of the study, 13 (4 males, 9 females) students in the case group had metabolic syndrome, which changed to 14 (10 males, 4 females) at the end of the second year. When components of MeS within the intervention group, were compared between the second year of study and at time of recruitment, results showed that although the number of overweight/obese individuals, individuals with hypertriglyceridemia and high LDL increased, abdominal obesity, high BP, hyperglycemia, and insulin resistance showed a significant decrease (p<0.001). In the control group, results showed that number of individuals with high BP increased significantly, however other MeS components did not show any significant change (table 4). The intervention group was 589 individuals at the beginning of the study, 5 of whom were excluded from the study at the end of the second year due to lack of access to anthropometric information. At the beginning of the study, blood tests were performed on 210 participants, and at the end of the second year, only 144 of them repeated the tests. The percentages were presented in total which McNemar test was performed between 117 individuals with identical valid data.

The control group was 803 individuals at the beginning of the study, which 592 of them had complete anthropometric information for analysis, and at the end of the second year, only 404 of them participated to measure the data. At the beginning of the study, blood tests were performed on 74 participants, and at the end of the second year, only 46 of them repeated the tests. The percentages were presented in total which McNemar test was performed between 34 individuals with identical valid data.

**Table 4:** Comparison of frequency distribution of metabolic syndrome components between intervention and control groups among elementary students at beginning of the study and end of the second year

**Variables**		**Timing of study**		**P-value**
		**At beginning**	**Second year**	
Abdominal obesity – no. (%)	Intervention	34 (5.8)	7 (1.2)	<0.001
	control	15(4.8)	15 (4.8)	1.000
Overweight – no. (%)	Intervention	4 (0.7)	42 (4.1)	<0.001
	control	34 (10.9)	24 (7.7)	
Obesity – no. (%)	Intervention	11 (1.9)	16 (2.7)	0.173
	control	39 (12.5)	43 (13.8)	
High blood pressure – no. (%)	Intervention	443 (76.4)	130 (22.4)	<0.001
	control	221 (72.2)	290 (94.8)	<0.001
High systolic blood pressure – no. (%)	Intervention	340 (58.5)	98 (16.9)	<0.001
	control	91 (29.7)	68 (22.2)	0.033
High diastolic blood pressure – no. (%)	Intervention	422 (70.7)	128 (22.0)	<0.001
	control	213 (69.6)	290 (94.8)	<0.001
Hypertriglyceridemia– no. (%)	Intervention	6 (5.1)	37 (31.6)	<0.001
	control	2 (5.9)	6 (17.6)	0.219
LDL >=110 mg/dL – no. (%)	Intervention	17 (14.5)	39 (33.3)	<0.001
	control	4 (11.8)	7 (20.6)	0.453
HDL– no. (%)	Intervention	38 (32.5)	30 (25.6)	0.215
	control	8 (23.5)	9 (26.5)	1.000
Hyperglycemia – no. (%)	Intervention	4 (3.3)	1 (0.8)	0.375
	control	-	-	-
Insulin resistance – no. (%)	Intervention	58 (27.4)	6 (9.5)	<0.001
	control	2 (6.1)	6 (18.2)	0.219
Metabolic syndrome – no. (%)	Intervention	7 (8.6)	13 (16)	0.180
	control	1 (4.8)	2 (9.5)	1.000

*Components of metabolic syndrome are defined according to the Program’s Adult Treatment Panel (ATP) III criteria as TG ≥ 150mg/dl, HDL-c < 40mg/dl, systolic blood pressure > 130mmHg or diastolic blood pressure > 85mmHg and FBS > 100mg/dl. †Insulin resistance was defined as insulin resistance index (HOMA-IR) greater than 2.1.

## Discussion

This study was part of a large prospective community-based study termed the "Sama" study which aimed to evaluate the effects of a comprehensive intervention on nutritional behaviors and physical activity on MeS components among students from elementary schools. Herein we found that the prevalence of overweight and obesity among elementary students was 16.7% and 18.9%, respectively. Abdominal obesity was prevalent in 5.2% of students, which is a proven risk factor for cardiovascular disease. Our findings showed a relatively high rate of overweight and obesity among elementary school children in an Iranian sample. In addition, a high percentage of hypertension and insulin resistance was observed in this population.

In a study by Ogden et al. (6) evaluating obesity among children between the ages of 2-19 years old during 2011 to 2012, they found that the prevalence of obesity was 17%. This was very similar to that of our study. Martins et al. (15) reported a prevalence of 13.1% and 9% for overweight and obesity among children of 7-11 years old from Brazil. Although exact comparison of studies is difficult due to the differences in study design and inclusion of different age groups, obesity is related to multiple factors including cultural, financial, social factors, geographical location and life style differences, thus emphasizing the importance of local studies to evaluate status of obesity in the region (7, 16).

In our study, after two years of intervention to increase function and awareness of students regarding lifestyle determinants, abdominal obesity in the intervention group decreased from 5.8% to 1.2%, however, the prevalence of overweight and obesity increased from 0.7% to 4.1% and from 1.9% to 2.7%, respectively. Interventional study of Franckle et al. was linked to a modest decrease in obesity rates among seventh-graders in one neighborhood, as well as improvements in behavioral targets, as compared to controls (17). Other studies have also found nutritional and physical activity interventions to be ineffective in reducing BMI (18, 19). Previous studies were not able to show a significant effect from school-based interventions on prevalence of obesity and overweight, this has been attributed to the short term intervention period and the lack of parental participation in children's intervention plans (20), however, in our study, we considered both teacher and parent roles in the lifestyle modification program. This shows that perhaps factors including the transition to puberty may have also affected the results of these studies.

Vos et al. (21) showed that lifestyle changes are ineffective in controlling obesity during short term and only affect obesity and over-weight during long follow-ups. This said, our findings did show that WC, which is among the most important risk factors for cardiovascular and metabolic diseases (22), to have a significant decrease in the intervention group. Insulin resistance was 22.3% among elementary students in our study. In the current study, after intervention, frequency of insulin resistance significantly decreased in the intervention group (27.4% to 9.5%), whereas in the control group, there was an increase in the prevalence of insulin resistance, which was not statistically significant. In the study by VOS et al. (21), after 2 years of lifestyle modification interventions, improvements were reported in HOMA-SDS values (21). In another study by Maiguis-Gray et al. in 2015 in Canada, 9 months of combined medication and HIIT program, significantly improved insulin resistance (23). These results show that the intervention program to improve lifestyle, at a cellular level in reducing insulin resistance, is more successful than reducing prevalence of overweight and obesity. Hyper insulinemia and insulin resistance are considered risk factors for the development of cardiovascular disease. In addition, it has a significant role in the development of MeS components, including lipid disorders and hypertension, so it is important to take preventive measures (24).

The rate of high blood pressure in our study among elementary school students was remarkable as we recorded 76.4% and 74.7% high blood pressure in our intervention and control groups, respectively. These rates were significantly higher than those recorded in previous literature from our region (25, 26). We used standard definitions, trained personnel and further considered sex and age for evaluating status of blood pressure in our study which presents more accurate results compared to previous literature.

Considering that in the intervention group high blood pressure had a significant decrease from 76.4% to 22.4% after intervention, this shows that by correcting dietary habits and omitting unhealthy snacks from children's diets and increasing daily physical activity, we can significantly decrease high blood pressure as a component of MeS among children in young ages. Similar results were seen in the study by Elizondo‐Montemayor et al. (27) in 2013. In their study, after a 10-month intervention program (which included an educational programs) for 6–12-year-old student, they found that high blood pressure decreased from 19% to 10%. Seemingly, lifestyle interventions seem to render different effects on blood pressure compared to body weight. 

In the present study, the intervention had no significant effect on the prevalence of MeS, which could be due to the low prevalence of MeS at this age. However, in a similar school-based study by Elizondo-Montemayor et al., the effects of low-calorie intake and increased physical activity for 10 months were evaluated on the prevalence of MeS in obese children aged 6 to 12 years. They found a significant decrease in the prevalence of MeS (From 44% to 16%) (27). The reason for discrepancy between studies is the lack of a standard definition and unequal cut-off points in anthropometric measurements, lipid levels, blood pressure and glucose, which makes it difficult to compare the prevalence of MeS among different studies. The study presents significant finding considering high rates of MeS in the Iranian population (28).

Before starting the intervention program, prevalence of other risk factors for cardiovascular diseases and MeS was evaluated in all elementary students. The prevalence of hyperglycemia was 2.4%, high LDL was 16.2%, low HDL was 31%, and high triglyceride was 5.3%. Among the elementary students, low HDL was the most common component of MeS and there was no significant difference between the two sexes in any of the above parameters. Caceres et al. found that the prevalence of MeS in individuals aged 5 to 18 years old in Bolivia was 36%, and low HDL (55.7%) was more common than other components of the MeS (hyperglycemia 8.2%, high triglyceride levels 42.6%) (29). After 3 years of lifestyle modification interventions, a significant increase was observed in high triglyceride rates (5.1% to 31.6%) and high LDL rates (14.5% to 33.3%), however prevalence of low HDL decreased from 32.5% to 25.6% and hyperlipidemia decreased from 3.3% to 0.8%, which was not statistically significant.

In a study by Elizondo-Montemayor et al., after interventions to improve school-based lifestyles in school children aged from 6 to 12 years, high triglyceride rates decreased from 64% to 35%, low HDL decreased from 60% to 41%, and hyperglycemia decreased from 1% to 0% (27).

The most important limitation of this study was the loss of participants to follow-up for reasons such as relocation. However, considering that this study is a community-based prospective trial, some lost to follow-up was expected. Furthermore, the three-year period chosen for the duration of the project is one of the longest intervention periods among the studies.

In conclusion we found that modifying dietary habits and increasing physical activities along with training increase the awareness of students and parents and making a change in their performance is a useful strategy for elementary school students that can significantly reduce some major components of MeS including high blood pressure and abdominal obesity, moreover, lifestyle modification interventions significantly decreased insulin resistance.
